# Intuitive Eating and the Female Athlete Triad in Collegiate Runners

**DOI:** 10.3390/nu17142337

**Published:** 2025-07-17

**Authors:** Janie Thomson, Hawley C. Almstedt

**Affiliations:** Department of Health and Human Sciences, Loyola Marymount University, Los Angeles, CA 90045, USA; janie.thomson.lmu@gmail.com

**Keywords:** disordered eating, dietary intake, eating behaviors, bone mineral density, osteoporosis, energy availability

## Abstract

**Background**: Female collegiate runners may be at high risk for disordered eating and poor bone health, which are characteristics of the female athlete triad. Intuitive eating can promote healthy eating behavior and adequate calorie intake, central variables in calculating energy availability, an underlying cause of low bone mass in athletes. Poor bone health can contribute to injury, preventing optimal performance for athletes. The purpose of this study was to assess intuitive eating, energy availability, and bone mineral density in female college runners with comparison to non-athletes. **Methods**: Female college athletes (*n* = 13, 19.5 ± 1.4 yrs) and non-athletes (*n* = 12, 19.9 ± 1.3 yrs) completed the Intuitive Eating Scale, Eating Disorder Examination Questionnaire, and menstrual history survey. Bone mineral density and body composition were measured using a dual-energy X-ray absorptiometer (DEXA). A 3-day diet record and exercise log were used to assess dietary intake, estimate energy expenditure, and calculate energy availability. **Results**: Intuitive eating was inversely correlated with disordered eating (*r* = −0.596, *p* = 0.002). Intuitive eating scores were not correlated to calorie intake, energy availability, bone mass, or percent body fat. Runners consumed significantly more calories, calcium, magnesium, phosphorus, and protein (g/kg) than non-athletes. Energy availability and bone mineral density were not significantly different between runners and non-athletes. **Conclusions**: Intuitive eating is associated with healthy eating behaviors in college-age females and was not related to energy availability, bone density, or body composition in this population. Future research could explore the use of intuitive eating principles in reducing disordered eating and addressing low energy availability in female runners and non-athletes.

## 1. Introduction

The female athlete triad is a syndrome composed of three interrelated disorders that occur on a spectrum of severity involving menstrual dysfunction, poor bone health, and low energy availability, with or without disordered eating [1]. More recently, a model termed relative energy deficiency in sport (REDS) expands concepts of the triad and its low energy availability, highlighting negative impacts beyond menstrual function and bone health to include cardiovascular, immune, and gastrointestinal physiology in both males and females [2].

Previous research has shown that disordered eating is related to low bone mineral density in female runners [3]. Athletes who are at risk for low energy availability also have a significantly higher risk for disordered eating [4]. Intuitive eating is an approach to eating behavior that encourages focusing on internal hunger and satiety cues, allowing the cues to guide food choices [5]. Restrictiveness and rigidity are common features of disordered eating. In contrast, the principles of intuitive eating encourage unconditional permission to eat, meaning that a person does not limit the type or quantity of food they consume based on external factors. Instead, intuitive eating encourages listening to internal signals from the body on what to eat, when to eat, and how much to eat so that a person feels satisfied but not overly full [5]. Previous research suggests that intuitive eating practices can be protective against disordered eating behaviors, and teaching someone to become a more intuitive eater may reduce disordered eating [6,7,8].

Among undergraduate college students, the incidence of disordered eating is 19.7% [9]. Female lean-sport athletes, like runners, are more likely to exhibit disordered eating behavior than other types of athletes [10]. Therefore, female collegiate runners are uniquely positioned to be at a higher risk for disordered eating. Disordered eating and high energy expenditure can contribute to the energy imbalance that is characteristic of low energy availability.

Energy availability, relative to lean mass, is a measure that represents the amount of energy the body has available to use after considering intentional exercise [11]. Low energy availability due to low caloric intake may compromise a person’s overall nutritional intake, including micronutrients important for bone health [3] and carbohydrates that are necessary for fueling exercise [4]. In particular, the inadequate intake of calcium, vitamin D, magnesium, and phosphorus may contribute to low bone mineral density [12].

Of major concern to female athletes is the increased risk for injury due to the low energy availability observed with triad/REDS. Of all injuries that occur in runners, 4.4–15.6% are skeletal stress fractures [13]. Female athletes are at a greater risk for stress fractures than their male counterparts, with an incidence rate of 9.7% among females compared to 6.5% in males [14], while other research suggests that the risk for females is at least twice that of males [15]. Risk factors for skeletal injury include large training volumes, low energy availability, menstrual dysfunction, and low bone mineral density (BMD) [15,16,17].

While the literature exists about the variables of energy availability, disordered eating, and bone health in female collegiate runners, intuitive eating has not been measured in relation to these variables. Therefore, the purpose of this research was to assess intuitive eating, energy availability, and bone health in female collegiate runners and non-athletic controls. We hypothesized that intuitive eating would be negatively correlated with disordered eating and energy availability. Additionally, we predicted that runners would have higher BMD at the hip when compared with non-athletes.

## 2. Materials and Methods

### 2.1. Design and Assessments

In this cross-sectional study, volunteers answered questionnaires online that collected information on demographics, menstrual function, and use of hormonal contraceptives. After completing the questionnaires digitally, the participants had an in-person testing appointment. Height and weight were each measured twice using a digital scale with a stadiometer (Health O Meter 500KL, McCook, IL, USA), and the mean value was determined. Grip strength was assessed three times on right and left sides using a digital dynamometer (Takei model T.K.K.5401, Kamo City, Niigata Prefecture, Japan) with 60 s of rest between each trial. The highest measurement for the right and left side was summed. The study protocol and informed consent documents were approved by the Loyola Marymount University Institutional Review Board and followed the Helsinki Declaration.

### 2.2. Participants

Runners (*n* = 13) were recruited from a National College Athletic Association Division I cross-country team through a presentation at a team practice and the distribution of flyers. Athletes trained 15–20 h per week with 2 days per week of resistance training and some cross-training such as swimming, cycling, or elliptical use. The non-athlete female controls (*n* = 12) were recruited through campus newsletters, flyers, and an in-class presentation. Inclusion criteria for controls were <300 min per week of moderate-to-vigorous physical activity and body mass index ≤ 25 kg/m^2^. Women who were pregnant or those who had a known metabolic bone disease were excluded from this study.

### 2.3. Intuitive Eating and Disordered Eating

Participants completed the 23-item Intuitive Eating Scale (IES-2), which produces a total score, as well as four subscale scores [18]. Possible values range from 1 to 5 with higher scores indicating greater use of intuitive eating principles. The subscales include unconditional permission to eat, eating for physical rather than emotional reasons, reliance on hunger and satiety cues, and body–food choice congruence. The IES-2 demonstrates more than acceptable reliability with Cronbach’s alpha reported at 0.87 and 0.88 for female populations in previous research [18].

Participants were asked to complete the previously validated Eating Disorder Examination Questionnaire (EDEQ) [19], which includes 28 items, scored between 0 and 6 for a total possible score of 6. The EDEQ is reported to have a high internal consistency with a Cronbach’s alpha of 0.93 [20]. The questionnaire provides a global score, as well as four subscale scores: dietary restraint, eating concern, shape concern, and weight concern. Varying cutoff values for eating disorder diagnosis were used in previous studies, but a global score of 1.24 aligns with the 50th percentile in previous research among adult females [21].

### 2.4. Bone Health and Body Composition

Dual-energy X-ray absorptiometry (DEXA; Hologic Horizon A, Apex Software 5.6.13, Waltham, MA, USA) was used to measure bone mineral density in both the athlete and non-athlete groups. Before their DEXA scan, participants were asked to remove any jewelry or metal that may contribute to the measurement of bone. Bone density was measured at the anterior–posterior spine, femoral neck, total hip, distal forearm, and whole body. The whole-body DEXA scan was used to measure percent body fat and bone-free lean mass (BFLM), the latter of which was used in the calculation of energy availability. Among athletes in weight-bearing sports, the American College of Sports Medicine (ACSM) defines low bone density as a z-score < −1.0 [1]. Repetitive DEXA scans in this facility have less than 1% coefficient of variation. One person operated the DEXA for all study participants. The bone-specific physical activity questionnaire (BPAQ) [22] was used to gather information about physical activity history, including all sports, ages of participation, and years of involvement.

### 2.5. Energy Availability and Dietary Intake

The variables of energy intake, energy expenditure, and bone-free lean mass were used to estimate energy availability using the equation below.

(1) $$\mathrm{Energy availability}=\frac{\mathrm{Energy intake}\left(\mathrm{kcal}\right)-\mathrm{Exercise energy expenditure}(\mathrm{kcal})}{\mathrm{Bone free lean mass}(\mathrm{kg})}$$

Participants were given a standardized diet log and were asked to record their diet for three typical days, including two weekdays and one weekend day. Visuals of standard portion sizes were provided to improve accuracy. Upon completing the 3-day diet record, the food log was reviewed by the primary researcher, and participants were interviewed to further clarify portion sizes and food details. Energy intake and dietary intake of nutrients important for bone health were determined from the 3-day diet records using the software Food Processor (ESHA Nutrition and Fitness Software version 11.14.9, Salem, OR, USA). Energy expenditure was calculated using a 3-day exercise log, which asked participants to record their exercise for the same three days as they recorded their diet. MET values were assigned to each exercise according to the 2011 Compendium of Physical Activities [23]. Activities with a MET value > 4 were included in the calculation of exercising energy expenditure. One athlete was unable to complete the 3-day diet record and exercise log; therefore, averages for 12 athletes are presented.

The Cunningham equation was used to estimate resting metabolic rate (RMR) and to determine what would have been the RMR during the time of exercise [24]. To improve the accuracy of exercising energy expenditure, similar to previous research, the RMR during exercise was subtracted from the calculation of energy expended during exercise to avoid double counting of these calories [25]. As mentioned earlier, DEXA was used to measure BFLM, the denominator in estimating energy availability. Previous research has established low energy availability to be <30 kcal/kg of BFLM per day, while reduced energy availability falls in the range between 30 and 44.9 kcal/kg BFLM [26].

### 2.6. Statistical Analysis

Statistical analysis was performed using the SPSS Statistics software version 29.01. Between-group differences were analyzed using independent-sample *t*-tests. An analysis of covariance was performed to test for differences between groups in bone mineral density variables while controlling for body mass (kg). Effect size was evaluated using Cohen’s *d* when significant differences were discovered. Pearson’s correlation coefficients were determined for the Eating Disorder Examination Questionnaire and Intuitive Eating Scale results. A *p*-value of <0.05 was used to determine statistical significance for all statistical tests. Effect sizes for significant group differences and strength of significant correlations were interpreted as large (≥0.8), medium (0.5–0.79), small (0.2–0.49), or nonexistent (0–0.19) [27].

## 3. Results

Runners and non-athletes were well-matched in age, height, BMI, BFLM, and grip strength (see Table 1). Most participants in both groups were white and not Hispanic. Independent samples t-tests revealed that body weight (*d* = 0.85), body fat percentage (*d* = 2.86), and fat mass (*d* = 2.40) were significantly higher in the control group than the athlete group. The grip strength of the athletes and non-athletes was similar, and grip strength was significantly related to bone mineral density at the anterior–posterior spine (*r* = 0.52, *p* < 0.01), femoral neck (*r* = 0.45, *p* < 0.02), and whole body (*r* = 0.49, *p* < 0.01). Grip strength was also significantly related to lean mass (*r* = 0.46, *p* < 0.02). Two participants reported oligomenorrhea, one of whom was an athlete and one of whom was not. Six participants, one athlete and five non-athletes, used oral contraceptives. Other forms of hormonal contraceptives were used by four participants, with an equal number of athletes and non-athletes.

**Figure 1:**
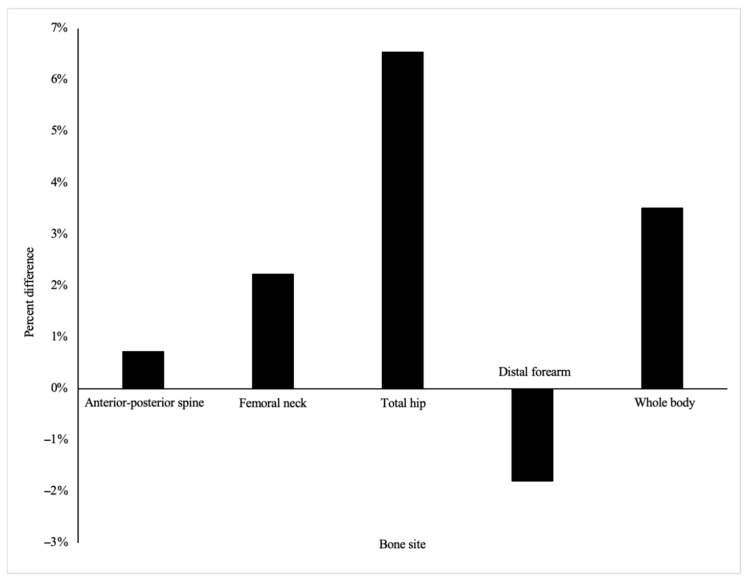
Percent difference in bone mineral density between female runners and non-athletes. Mean bone density is not significantly different between athlete and non-athlete groups.

When controlling for body weight, the bone mineral density of athletes and non-athletes was similar at all sites (Table 2). Although not significantly different, athletes had greater BMD at all bone sites, except the forearm, where the control group had 1.8% greater BMD (Figure 1). The BMD for runners and controls was not different when tested with or without body weight as a covariate. One athlete and two non-athletes had low BMD, indicated by a whole-body z-score of ≤−1.0. At the anterior–posterior spine, three athletes and three non-athletes had low BMD. No participants had low BMD at the femoral neck or total hip, but the Hologic DEXA did not report hip z-scores for three athletes and four non-athletes due to a lack of normative data. Mean z-scores of whole-body BMD were significantly higher in athletes (−0.11 ± 0.61) than non-athletes (−0.60 ± 0.59, *p* = 0.05, *d* = 0.82). Runners demonstrated significantly greater current bone-loading physical activity measured via the BPAQ (*d* = 0.77). However, past and total BPAQ measures were similar between groups.

Runners consumed significantly more calories (*d* = 1.41), carbohydrates (*d* = 1.44), protein (*d* = 1.13), calcium (*d* = 1.09), magnesium (*d* = 1.09), and phosphorus (*d* = 1.18) than non-athletes. Levels of vitamin D intake were also higher in athletes than non-athletes (*d* = 0.39), though the difference was not statistically significant. Most athletes and non-athletes did not meet the Recommended Dietary Allowance (RDA) for micronutrients that are key for bone health. However, of the participants who did consume the RDA of each micronutrient, most were athletes. A majority of participants (75%) did not meet the RDA for vitamin D [28]. More specifically, 33% of the athlete group met the RDA for vitamin D, compared to 17% in the non-athlete group. Many participants did not meet the RDA for calcium or magnesium, with only 8.3% meeting the recommendation for these two minerals, all of whom were athletes [28]. Phosphorus intake was appropriate for age in 23% of volunteers, with 42% of athletes and 17% of the non-athletes meeting the RDA [28]. On average, athletes and non-athletes ate sufficient protein for optimal sports nutrition [29]. While runners ate significantly more carbohydrates than controls, they still fell below the 6–10 g/kg recommendation for endurance-trained athletes [29].

No significant difference in energy availability was found between runners and non-running controls. Of the participants who met the criterion for reduced energy availability, 75% were non-athletes. Of the participants whose energy availability was classified as low energy availability, 50% were non-athletes. Energy availability was not correlated to bone mineral density at any site. As shown in Table 3, non-running controls exhibited significantly higher levels of unconditional permission to eat than athletes (*d* = 0.88), though athletes had greater body–food choice congruence than non-athletes (*d* = 0.92). Table 4 displays many statistically significant correlations between intuitive eating and disordered eating, including the four subscales for each questionnaire. Unconditional permission to eat from the IES-2 had a strong (*r* > 0.8) negative relationship with the global score, dietary restraint, and eating concern from the EDEQ. Moderately strong negative relationships (*r* = 0.5–0.79) were also found between the IES-2 global score and the EDEQ global score as well as three of the four EDEQ subscales. Unconditional permission to eat had a moderately strong negative relation with shape concern and weight concern, while reliance on hunger/satiety cues had a moderately strong negative relationship with the global EDEQ, eating concern, and shape concern. Intuitive eating was not found to be correlated with calorie intake, energy availability, bone mineral density, fat mass, or body fat percentage.

## 4. Discussion

A primary finding from this study is that intuitive eating is inversely correlated with disordered eating. The magnitudes of the negative relationships between variables of the IES-2 and EDEQ appear to be moderate (*r* = 0.5–0.79) or strong (*r* > 0.8). Previous studies also report negative correlations between intuitive eating practices and disordered eating [15,16,17]. This finding contributes to the validity of the Intuitive Eating Scale, suggesting that it measures what it seeks to measure. Further, it supports the concept that practicing intuitive eating may be protective in preventing disordered eating [8,30]. The moderately strong negative correlation between the global score of disordered eating and the global score of intuitive eating suggests that the more a person practices intuitive eating, the lower their disordered eating score. In reviewing the disordered eating global score with unconditional permission to eat, a strong negative correlation indicates that people with high scores for unconditional permission to eat also have low disordered eating. A strong negative correlation was found between unconditional permission to eat and dietary restraint, suggesting that a person giving themselves unconditional permission to eat is protective against behaviors of dietary restraint. These findings suggest promoting intuitive eating to foster healthy dietary habits for athletes at risk for disordered eating and the female athlete triad/REDS.

Previous research suggests that intuitive eating is associated with lower BMI [31] and lower percent body fat [32]. However, we did not find intuitive eating to be related to BMI or body fat percentage in this population of college-age females, which is a particularly important finding because people may be hesitant to eat intuitively because they worry that it will negatively affect their body composition. For athletes, the concern that their performance may be affected by their body fat percentage could especially be a barrier to intuitive eating. Practicing intuitive eating may be helpful in reducing restrictive eating patterns, psychological distress, and improving mental health [7,8] even if there is no obvious association with body fat, energy availability, or BMD. Notably, on average, athletes displayed a nonsignificant 9 kcals/kg more energy availability than non-athletes. This was somewhat surprising given the athletes’ high levels of energy expenditure and frequent reports of low energy availability among athletes in previous research. Considering the moderate to strong negative correlations between intuitive eating and disordered eating, one might assume that intuitive eating would also be correlated with energy availability; however, this was not the case in the current study. Overall, these runners showed healthy energy availability, whereas there were more non-running controls with reduced or low energy availability. This is an interesting finding because energy availability in college-age, non-athlete populations is not frequently reported in the existing literature. Perhaps the lack of relationship between intuitive eating and energy availability reported here is due to the generally appropriate matching of calorie consumption to exercise activity among runners in this study or the imprecise nature of assessing energy availability.

Further, the data did not reveal significantly different bone mineral density between the runners and non-runners. This was also an unexpected finding because running is a weight-bearing sport, especially at the hip, which should promote higher levels of bone density among athletes [33]. Goodwin et al. reported that most (80%) of the elite Kenyan runners and age-matched controls in their study had low bone mineral density, particularly at the spine [34]. Another investigation of American high school runners found that non-running controls had a higher prevalence of low BMD than female runners [35]. We report very few participants with low bone mineral density, and, of those that were low, they were evenly distributed between athlete and non-athlete groups. Goodwin et al. did not find statistically significant differences between the BMD of the athletes and non-athletes at the lumbar spine or the femoral neck [34]. This finding aligns with the data we collected, since athletes and non-athletes were not statistically different in BMD at any site.

While the non-athletes in this study were significantly less active than runners at the time of data collection, their past bone-loading physical activity was similar (see Table 1). Knowing that weight-bearing physical activity undertaken near the adolescent growth spurt can have long-term impacts on bone health [36], it is possible that past bone-loading activity among controls in this study led to a similar BMD between athletes and non-athletes at nearly the age of 20. Also, as seen in Figure 1, it is likely that the weight-bearing nature of running provides an advantage in BMD at the hip and whole body, while the specificity of training creates less of an advantage at the spine and forearm compared to controls. This concept is supported by previous research reporting similar or lower BMD at the spine in female runners compared to controls [37,38]. Further, the use of oral contraceptives may have influenced BMD among participants in this study, as one runner and five controls reported use of the hormone-containing medications. Previous research reports lower BMD among oral contraceptive users; however, this influence may depend on dose, type of progesterone, years of use, and age when the initiation of medication began [39,40].

One of the few studies that has compared the energy availability of athletes to non-athletes found that both groups had a high prevalence of low energy availability, with 69% of participants having reduced energy availability [34]. In contrast, 42% of participants in our study were found to have reduced energy availability. Both studies compared female runners and non-runners, with the study conducted by Goodwin et al. [34] assessing participants 18–30 years old, while our study’s participants had a narrow age range of 18–23. Differences in the prevalence of low energy availability may be due to the method of measuring energy intake. Goodwin et al. weighed the participants’ food and used different software to calculate energy intake [34]. In comparison, we used a 3-day diet record protocol, which was analyzed using Food Processor software by a trained researcher.

In comparing female high school athletes to non-athletes, one study found that the two groups had similar percentages of participants with reduced energy availability (36% for athletes vs. 39% for non-athletes) [35]. Our study found that there were more non-athletes with reduced/low energy availability than athletes (25% for athletes vs. 58% for non-athletes). While the proportion of reduced/low energy availability was surprisingly larger among non-athletes, the mean energy availability for the two groups in this study was not statistically different.

Overall, we found that many of the female college students in this study did not have a sufficient intake of micronutrients that are important for bone health. The adequate intake of bone nutrients is essential for optimal health, athletic performance, preventing skeletal injuries, and promoting recovery when injury does occur. The sufficient intake of calcium and optimal vitamin D status are especially important for bone health and should be emphasized as part of a comprehensive and balanced diet [1,12]. Only 8.3% of participants in our study met the RDA for calcium, with those participants exclusively being athletes. In contrast, a previous study on female collegiate runners found that 50% of the participants did not meet the RDA for calcium [41]. Similar to the Beermann et al. study, most of our participants (75%) also failed to meet the RDA for vitamin D [41]. In addition, large portions of our research participants did not meet the RDA for either magnesium or phosphorus (92% and 77%, respectively), most of whom were non-athletes. Failure to meet the RDA for these micronutrients puts someone at greater risk of skeletal injury and may lead to suboptimal bone accrual in young people [1]. In general, the athletes had better adherence to the RDAs for bone nutrients than the non-athletes, suggesting that they may have healthier dietary intake. Runners in this study consumed significantly more carbohydrates and protein than non-athletes; however, they still fell short of carbohydrate recommendations needed for optimal performance in a high-intensity endurance sport [29].

### Strengths and Limitations

Despite using valid methods employed in previously published research, diet records will never perfectly measure dietary intake. Further, 3-day diet records reflect a limited span of a person’s diet and therefore may not fully represent long-term nutrition [42]. This may be particularly true for athletes, whose competitive schedule can involve fluctuation in training and nutritional needs. All methods of assessing dietary intake have their strengths and weaknesses; however, the 3-day diet record approach is commonly used in estimating energy availability [43]. Recording diet and accurately estimating portion sizes can be challenging for participants, and the nutritional makeup of foods consumed by the participants may not always be accurately reflected by the Food Processor software. Further, unique or less popularly consumed foods may not be in the software database, making it challenging to represent the exact nutritional content of foods consumed.

This study has clear strengths as well as limitations. To our knowledge, this is the first study to investigate intuitive eating in relation to components of the triad/REDS. The DEXA was used to evaluate BMD, which is considered the gold-standard protocol [44]. Bone density was measured at several sites, providing a complete picture of overall bone health. However, Hologic, the manufacturer of the DEXA machine involved in this study, uses data from the Centers for Disease Control (CDC) National Health and Nutrition Examination Survey. This system does not provide z-scores for bone density at the femoral neck or total hip of young Hispanic women, and one-third of the participants in this study identified as Hispanic. The implication for this study was that the z-score data at the hip sites for Hispanic participants were not available, limiting the comprehensive nature of our analysis. The CDC should work to establish BMD norms for women of all races/ethnicities to be incorporated by Hologic.

Extrapolation of these research findings is limited by the small sample size. Despite reporting no significant differences in BMD between athletes and non-athletes in this study, examination of the Cohen’s d effect size as small to medium for all bone sites suggests that this study was underpowered, which limits generalizability and extrapolation. Because data collection took place over several weeks, some of the female runners were in post-season, while others were continuing to compete, which may have influenced the measurement of energy expenditure and intake. It would be ideal to assess energy availability at the peak of the season for all runners, when their training is at its highest intensity. Doing so would help standardize the runners’ measures of energy balance.

## 5. Conclusions

Energy availability and bone health are key aspects of the female athlete triad and REDS. Intuitive eating is associated with healthy eating behaviors in college-age runners, as well as in non-athlete females, and does not appear to be related to body composition. Gaps in the micronutrient intake of female college students suggest room for improvement to support optimal bone health. Further education about nutrients important for bone health and behavior change methods to incorporate these nutrients is needed among college-age women. Coaches, registered dietitian nutritionists, and sports medicine professionals should collaborate to ensure adequate energy intake, which achieves sufficient energy availability, given the exercise energy expenditure for athletes. A logical next step is to implement an intuitive eating intervention among female athletes experiencing the triad or REDS. Future research should continue to explore intuitive eating in relation to bone health and energy availability, as well as widen the examination of these variables in non-athlete female populations. More research is needed to explore whether there are health consequences of low energy availability in non-athletes.

## Figures and Tables

**Table 1:** Participant characteristics (mean ± SD or number, %). Values in **blue** font indicate significant differences.

Variables	Runners (*n* = 13)	Controls (*n* = 12)	*p*-Value
Age (years)	19.5 ± 1.4	19.9 ± 1.3	0.491
Height (cm)	164.8 ± 6.6	165.3 ± 4.8	0.836
Body weight (kg)	58.7 ± 4.8	63.1 ± 5.6	0.045
Body mass index (kg/m^2^)	21.7 ± 2.2	23.1 ± 2.0	0.109
Body fat (%)	27.6 ± 2.0	34.8 ± 3.0	<0.001
Fat mass (kg)	16.6 ± 2.2	22.5 ± 3.4	<0.001
Bone-free lean mass (kg)	41.3 ± 3.6	40.1 ± 3.6	0.379
Grip strength (kg)	52.6 ± 8.5	49.0 ± 7.3	0.273
Bone-loading physical activity:			
Current	8.1 ± 3.2	3.7 ± 7.6	0.033
Past	59.4 ± 57.0	60.3 ± 56.2	0.484
Total	33.8 ± 29.3	32.0 ± 28.8	0.440
Race:			
Asian	0, 0%	1, 8.3%	
Black/African American	2, 15.4%	1 8.3%	
Native Hawaiian/Pacific Islander	1, 7.7%	0, 0%	
White	10, 76.9%	9, 75.0%	
Decline to state	0, 0%	1, 8.3%	
Ethnicity:			
Hispanic	3, 23.1%	5, 41.7%	
Not Hispanic	10, 76.9%	7, 58.3%	

**Table 2:** Bone mineral density (g/cm^2^) at various skeletal sites (mean ± standard error).

Bone Site	Runners (*n* = 13)	Controls (*n* = 12)
Spine	0.982 ± 0.025	0.975 ± 0.026
Femoral neck	0.873 ± 0.022	0.854 ± 0.023
Total hip	1.009 ± 0.026	0.947 ± 0.027
Radius and ulna	0.548 ± 0.010	0.558 ± 0.010
Whole body	1.090 ± 0.014	1.053 ± 0.015

**Table 3:** Daily nutrient intake, energy availability, intuitive eating, and disordered eating (mean ± SD). Values in blue font highlight significant differences between groups.

Nutrients	Runners	Controls	*p*-Value
Energy availability (kcals/kg)	52.0 ± 13.4	43.4 ± 9.1	0.084
Calories (kcal)	2465.0 ± 509.7	1853.2 ± 338.4	0.002
Carbohydrates (g/kg)	4.96 ± 0.98	3.48 ± 0.72	<0.001
Protein (g/kg)	1.75 ± 0.63	1.20 ± 0.30	0.021
Vitamin D (mcg)	9.68 ± 7.55	6.07 ± 10.70	0.350
Calcium (mg)	910.4 ± 320.0	575.9 ± 296.5	0.014
Magnesium (mg)	217.9 ± 88.4	125.6 ± 80.7	0.014
Phosphorus (mg)	913.1 ± 450.2	507.4 ± 179.3	0.008
Intuitive Eating Global Score	3.53 ± 0.65	3.71 ± 0.55	0.459
Unconditional Permission to Eat Subscale	3.38 ± 0.83	3.99 ± 0.49	0.039
Eating for Physical Rather Than Emotional Reasons Subscale	3.24 ± 0.78	3.61 ± 0.63	0.218
Reliance on Hunger/Satiety Cues Subscale	3.70 ± 0.92	3.74 ± 0.84	0.929
Body–Food Choice Congruence Subscale	4.26 ± 0.56	3.42 ± 1.18	0.031
Disordered Eating Global Score	1.31 ± 1.33	0.92 ± 0.65	0.366
Dietary Restraint Subscale	0.78 ± 1.08	0.28 ± 0.36	0.140
Eating Concern Subscale	0.95 ± 1.26	0.46 ± 0.50	0.223
Shape Concern Subscale	1.86 ± 1.67	1.49 ± 0.99	0.513
Weight Concern Subscale	1.66 ± 1.63	1.46 ± 1.29	0.746

**Table 4:** Bivariate relationships between disordered eating and intuitive eating (*n* = 25).

	1	2	3	4	5	6	7	8	9	10
Intuitive Eating Global Score (1)	1	**0.728**	**0.803**	**0.877**	**0.438**	**−0.596**	**−0.590**	**−0.626**	**−0.580**	**−0.421**
Unconditional Permission to Eat Subscale (2)		1	**0.468**	**0.576**	−0.042	**−0.814**	**−0.867**	**−0.829**	**−0.691**	**−0.652**
Eating for Physical Rather Than Emotional Reasons Subscale (3)			1	**0.514**	0.161	−0.350	**−0.427**	−0.325	**−0.448**	−0.129
Reliance on Hunger/Satiety Cues Subscale (4)				1	**0.443**	**−0.571**	**−0.476**	**−0.648**	**−0.518**	**−0.459**
Body–Food Choice Congruence Subscale (5)					1	0.135	0.227	0.105	0.124	0.074
Disordered Eating Global Score (6)						1	**0.840**	**0.936**	**0.917**	**0.928**
Dietary Restraint Subscale (7)							1	**0.808**	**0.703**	**0.656**
Eating Concern Subscale (8)								1	**0.779**	**0.847**
Shape Concern Subscale (9)									1	**0.795**
Weight Concern Subscale (10)										1

Values in **bold** indicate *p* < 0.05. Values in **blue** font highlight strong relationships (*r* > 0.80) between intuitive eating variables and measures of disordered eating, while **green** font indicates moderate (*r* = 0.5–0.79) relationships.

## Data Availability

The raw data supporting the conclusions of this article will be made available by the corresponding author upon reasonable written request.

## Abbreviations

The following abbreviations are used in this manuscript:

BFLM	Bone-free lean mass
BMD	Bone mineral density
BMI	Body mass index
BPAQ	Bone-loading physical activity questionnaire
CDC	Centers for Disease Control
DEXA	Dual-energy X-ray absorptiometry
EDEQ	Eating Disorder Examination Questionnaire
IES	Intuitive Eating Scale
RDA	Recommended Dietary Allowance
REDs	Relative energy deficiency in sport
RMR	Resting metabolic rate
